# Deep neural networks for automated detection of marine mammal species

**DOI:** 10.1038/s41598-020-57549-y

**Published:** 2020-01-17

**Authors:** Yu Shiu, K. J. Palmer, Marie A. Roch, Erica Fleishman, Xiaobai Liu, Eva-Marie Nosal, Tyler Helble, Danielle Cholewiak, Douglas Gillespie, Holger Klinck

**Affiliations:** 1 Center for Conservation Bioacoustics, Cornell Lab of Ornithology, Cornell University, Ithaca, NY 14850 USA; 2 Department of Computer Science, San Diego State University, San Diego, CA 92182 USA; 3 Department of Fish, Wildlife and Conservation Biology, Colorado State University, Fort Collins, CO 80523 USA; 4 Department of Ocean and Resources Engineering, University of Hawai’i at Mānoa, Honolulu, HI 96822 USA; 5 US Navy, Space and Naval Warfare Systems Command, System Center Pacific, San Diego, CA 92152 USA; 6 Northeast Fisheries Science Center, National Marine Fisheries Service, National Oceanic and Atmospheric Administration, Woods Hole, MA 02543 USA; 7 Sea Mammal Research Unit, Scottish Oceans Institute, University of St. Andrews, St Andrews, Fife KY16 8LB Scotland

**Keywords:** Animal migration, Conservation biology, Computer science

## Abstract

Deep neural networks have advanced the field of detection and classification and allowed for effective identification of signals in challenging data sets. Numerous time-critical conservation needs may benefit from these methods. We developed and empirically studied a variety of deep neural networks to detect the vocalizations of endangered North Atlantic right whales (*Eubalaena glacialis*). We compared the performance of these deep architectures to that of traditional detection algorithms for the primary vocalization produced by this species, the upcall. We show that deep-learning architectures are capable of producing false-positive rates that are orders of magnitude lower than alternative algorithms while substantially increasing the ability to detect calls. We demonstrate that a deep neural network trained with recordings from a single geographic region recorded over a span of days is capable of generalizing well to data from multiple years and across the species’ range, and that the low false positives make the output of the algorithm amenable to quality control for verification. The deep neural networks we developed are relatively easy to implement with existing software, and may provide new insights applicable to the conservation of endangered species.

## Introduction

Detecting animals that cannot readily be observed visually is a perennial challenge in ecology and wildlife management. Technological advances have led to development of detection methods such as environmental DNA (eDNA)^1–3^, cameras with infrared sensors and triggers (camera traps)^4,5^, chemical sensors (electronic^6^ and biological^7^) and satellite images^8^. In both marine ecosystems and terrestrial ecosystems, passive acoustic systems have been used to detect and monitor taxonomic groups that communicate by sound. Examples include but are not limited to cetaceans^9^, passerines^10^, chiropterans^11,12^, anurans^13^, orthopterans^14^, and proboscids^15^.

Passive acoustic monitoring (PAM) to detect the vocalizations of animals in real time or in archival data typically uses a combination of automated or semi-automated computer algorithms and manual verification by human analysts to assess animal presence, vocal activity, and behaviors such as breeding or foraging^16^. PAM also has been integrated with standard ecological sampling or analysis methods such as distance sampling^17^ and occupancy modelling^18^ to estimate habitat use and, in limited circumstances, animal abundance^19–21^. Moreover, long-term and spatially extensive collection of passive acoustic data may allow inference to whether environmental changes, including increases in ambient sound levels, affect the distributions or activities of marine taxa^9,22,23^.

Over the last decade, the cost of collecting and storing acoustic data has fallen dramatically and terabytes of data may be collected in a single project^24,25^. As the volume of acoustic data increases, it becomes more costly and time consuming to extract meaningful ecological information.

Machine learning has the potential to identify signals in large data sets relatively cheaply and with greater consistency than human analysts^26^. Methods such as discriminant analysis^27^, Gaussian mixture models^28^, support vector machines^29^, classification and regression trees^30^, random forests^31^, sparse coding^32^, and deep learning^32–36^ have been used in acoustic monitoring.

Deep learning refers to many of the advances in artificial neural networks over the last decade^37^. These advances include training of neural networks that have many layers, the ability to discover features that improve signal discrimination, and the availability of large data sets that allow proper training of the neural network models^38,39^. Here, we investigate the ability of deep neural networks to extract biologically meaningful information from large sets of passive acoustic data. Our work differs from published deep network approaches in terms of the task and scope. We identify individual calls as opposed to periods of time during which animals were present and calling^34^, and apply neural networks to data that are more geographically extensive than those presented by others^33–35^. We also investigate the ability of models trained with data from a small subset of the range of a highly migratory marine mammal to generalize across data collected throughout the species’ range and to detect rare calls within a long time series. Such a temporally and spatially comprehensive analysis can inform strategies for monitoring threatened and endangered species with large ranges. Few existing studies address the detection of calls over extended periods of time. Many studies either omit signal-absent cases or do not train detectors on representative sounds in which the target signal is absent. As a result, these studies do not provide a realistic estimate of how often the detector produces false positives, which can be triggered by diverse sources and propagation conditions.

As a proof of concept, we apply deep neural methods to the detection of a stereotyped contact call, the upcall, produced by North Atlantic right whales (*Eubalaena glacialis*)^40^. North Atlantic right whales (hereafter, *right whales*) are listed as endangered under the U.S. Endangered Species Act and by the International Union for Conservation of Nature^41^. Although we use the right whale as an example, our methods are transferable to other species in different acoustic environments.

Right whales occupy the western Atlantic Ocean from southern Greenland and the Gulf of St. Lawrence south to Florida. However, occurrence and movements of the species within some parts of their range^42^, such as the waters off the US coast between Georgia [32°N] and Cape Cod [42°N] and west of the Great South Channel [41°N, 69°W], is not well known. The population had been recovering, but has declined in recent years^43^. In 2017, an unusual mortality event resulted in the loss of 17 individuals, and fewer than 450 individuals are estimated to be extant^44^.

Limited knowledge about the location and movements of right whales hinders efforts to manage human activities aimed at minimizing mortality and preserving of habitat quality. Collisions with ships and entanglement in fishing gear are the principal sources of mortality and sub-lethal injury of right whales^45–47^, and current mortality rate is depressing the ability of this long-lived (70 a) species, which has low reproductive rates, to recover^48^. In addition, anthropogenic underwater sounds may increase levels of stress in marine mammals, including right whales^49^, and decrease their ability to communicate with conspecifics, find prey, or detect and evade predators^22^. Furthermore, some measures to protect right whales may have, or are perceived to have, negative effects on economic activities (e.g., shipping and commercial fishing), national defense (e.g., restrictions on conducting sonar training exercises), and energy development (e.g., construction and operation of offshore wind farms). Accordingly, there is considerable practical value in the development of robust detection and monitoring systems for the species.

Both sexes and all age classes of right whales produce upcalls^50,51^, which are used as a proxy measure of their presence^51–53^. Because of their stereotyped nature, upcalls often are the target of detection by acoustic monitoring systems for the species^53–55^. However, the likelihood of detecting bioacoustic signals varies among locations, environmental conditions and recording instruments^56^. It is affected by differences in vocal behavior of individual animals, or of the same animal at different times and locations^50^. Because right whales have been studied extensively over the past decades, a substantial archive of calls is available and can be used to train and test deep learning systems. We use data provided by the National Oceanic and Atmospheric Administration’s Northeast Fisheries Science Center for the 2013 workshop on the Detection Classification, Localization, and Density Estimation of Marine Mammals (DCLDE 2013^57^) and data collected by Cornell University’s Bioacoustics Research Program (BRP, now Center for Conservation Bioacoustics) from 2012–2015 (Table 1) to train and test deep neural network-based detectors (henceforth *deep nets*).

**Table 1:** Data sources used to train and evaluate deep neural network performance.

	Recording date	Region	Contract/Grant	Number of recorders	Total Recording Hours	Number of upcalls
DCLDE 2013 workshop	28-Mar-09	Massachusetts	i.^78–80^	1	24	767
	29-Mar-09	Massachusetts	i.^78–80^	1	24	2,280
	30-Mar-09	Massachusetts	i.^78–80^	1	24	1,663
	31-Mar-09	Massachusetts	i.^78–80^	1	24	2,206
	1-Apr-09	Massachusetts	i.^78–80^	1	24	1,328
	2-Apr-09	Massachusetts	i.^78–80^	1	24	545
	3-Apr-09	Massachusetts	i.^78–80^	1	24	894
2012-2015 MARU deployments	6-Sep-12	Georgia	ii.^81^	3	72	1
	14-Oct-12	Georgia	ii.^81^	3	72	118
	29-Dec-12	North Carolina	ii.^81^	3	72	12
	12-Mar-14	Virginia	iii.^82^	5	120	8
	25-Jan-15	Maryland	iv.^83^	9	216	448
	24-Jul-15	Maryland	iv.^83^	10	240	14
Kaggle	Massachusetts					7,027 (22,973 negative examples)

Number of upcalls indicates the number of upcalls annotated by trained analysts. For deployments with two or more recorders, the number of upcalls indicates the total number of upcalls detected across all recorders. Shaded rows indicate data used to train neural networks. Non-shaded rows represent evaluation data. Negative examples for the Kaggle data represent the false detections flagged by the analysts as derived from non-right whale sources. Contract grants: (i) Office of Naval Research grant (number N00014–07-1–1029) awarded by the National Oceanographic Partnership Program; (ii) U.S. Department of the Interior, Bureau of Ocean Energy Management grant (number M10PC00087); (iii) U.S. Department of the Interior, Bureau of Ocean Energy Management grant (number M15AC00010); (iv) U.S. Department of the Interior, Bureau of Ocean Energy Management grant (number M14AC00018); Maryland Department of Natural Resources grants (14-14-1916, 14-17-2241).

The DCLDE 2013 data contain calls from both right whales and humpback whales (*Megaptera novaeangliae*), both of which inhabit the coastline of the eastern United States. The species co-occur during spring, when their habitat and migratory routes overlap. Humpback whales produce many different types of calls^58^, and the songs and distribution of call types vary among years. One humpback note is sufficiently similar to the right whale upcall to be a major challenge for detection algorithms. Therefore, the degree of spatial overlap and call similarity may be high during a year in which an upcall-like note is present in the humpback whale song and quite low in another year in which it is not.

In applying deep nets to a conservation need, we addressed three main objectives. First, we explored whether deep nets yield greater precision and recall than currently deployed right whale upcall detectors. In this case, precision is the proportion of output detections that correspond to verified right whale upcalls, and recall is the proportion of verified right whale upcalls in the data that are identified by the detection algorithm. Second, we assessed the extent to which deep nets that were trained with data from one geographic region and season detected upcalls collected across multiple regions, seasons, and years (i.e., generalized across data sets). Third, we evaluated whether deep nets can produce good recall rates with number of false positives low enough to make analyst verification of detections feasible. Throughout this manuscript we refer to the deep neural networks as detection algorithms. However, detectors are binary classifiers, and therefore either term is appropriate.

## Results

### Comparison of deep neural networks and current detection methods

To compare the performance of the deep nets to that of currently implemented algorithms, we measured precision, recall, and the number of false positives per hour generated by our neural networks and by the systems presented by participants in the DCLDE 2013 workshop. We followed the DCLDE 2013 workshop protocol for selecting training and testing data and built and trained five deep nets on the basis of methods described in LeCun and Bengio^38^, Kahl, *et al*.^34^, Xu, *et al*.^59^, Simonyan and Zisserman^60^, and He, *et al*.^61^ with the minor modifications described in the methods. We used the training data (the first four days of continuous sound recordings) to develop binary classifiers that discriminate between the positive class (right whale upcalls) and the negative class (other sound sources), and evaluated the detection performance with the testing data, the final three days of recordings.

The algorithms presented at the DCLDE 2013 workshop used the same training and testing partition of the data. The classifiers used handcrafted features and a variety of traditional machine learning techniques such as: multivariate discriminant analysis^53^, generalized likelihood ratio tests^62^, decision trees^63^, shallow neural networks^64^, and boosting classifiers^65^.

**Figure 1:**
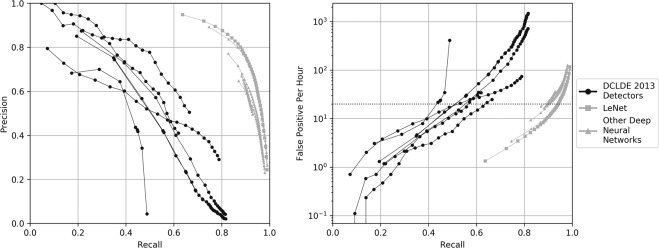
(Left) Comparison of precision and recall between the anonymized DCLDE 2013 results and those of the deep nets we evaluated. (Right) Average number of false positives per hour generated by application of different detectors to the DCLDE 2013 testing data (72 h) as a function of recall. The grey dotted line at 20 false positives per hour represents the maximum acceptable number of false-positive detections in a quality control process.

The performance of our five deep nets varied, but all yielded considerably higher precision and recall, and fewer false positives, than the algorithms presented at the DCLDE 2013 (Fig. 1). The outputs of the five upcall detectors we tested were similar. However, the deep net based on LeNet had the highest precision and lowest number of false positives for a given recall. BirdNet performed nearly as well as LeNet but had a higher computational cost due to a larger number of parameters and a complex network architecture. None of the DCLDE 2013 detectors produced recall rates above 0.83. At recall rates above 0.6, the precision of the DCLDE 2013 detectors dropped considerably. In contrast, deep nets had roughly double the precision and much improved recall than the best of the DCLDE 2013 detectors. Although precision captures the number of false positives, it does not effectively characterize the frequency with which they occur. Therefore, we also present the recall relative to false positives per hour, which is relevant to system verification. Across the overlapping recall range, the deep net architectures produced an order of magnitude fewer false positives per hour than all DCLDE 2013 detectors.

### Temporal and spatial generalization performance

The DCLDE 2013 data contained a high number of recorded and annotated upcalls that are useful for evaluating the precision and recall of diverse models. However, these data were collected over a single week in Massachusetts Bay. Therefore, these data represent only a small sample of the individuals, behavioral states, and environmental conditions that occur throughout the right whales’ range. We evaluated the generalization performance in six different coastal regions from Maryland to Georgia, along the migratory route of right whales. These data represent 33 days of recording effort on six different days during three-year period (2012–2015) (Table 1). During migration, right whales are less likely to remain in an area for a long period of time and also less likely to call, a behavior that may be related to avoiding predation^66^. As a consequence, the number of upsweeping calls recorded and annotated in the 33 days of analysis effort was limited to 601 calls. Given that DCLDE 2013 detector results were not available for these data, we compared our results to those from a baseline detector^64^ that was evaluated in the DCLDE 2013 workshop. We used default settings to test generalization of the baseline detector, although operators typically adapt the baseline detector’s parameters to specific data sets. Because the deep nets results tended to cluster, we used a single architecture for this step. We used the deep net architecture derived from LeNet for generalization testing because it performed best in the Massachusetts Bay environment.

The baseline detector was trained with data from a Kaggle data competition. To differentiate the gain in classifier performance attributable to the deep net architecture from that attributable to the use of different training data, we trained one deep net with the Kaggle data. The Kaggle data consist of right whale detections and false positives from an earlier detector^53^. Therefore, any detector that learns features is unlikely to generalize equally well or better because it is unlikely to have access to diverse signal-absent training examples. We trained a second deep net with the DCLDE 2013 data.

**Figure 2:**
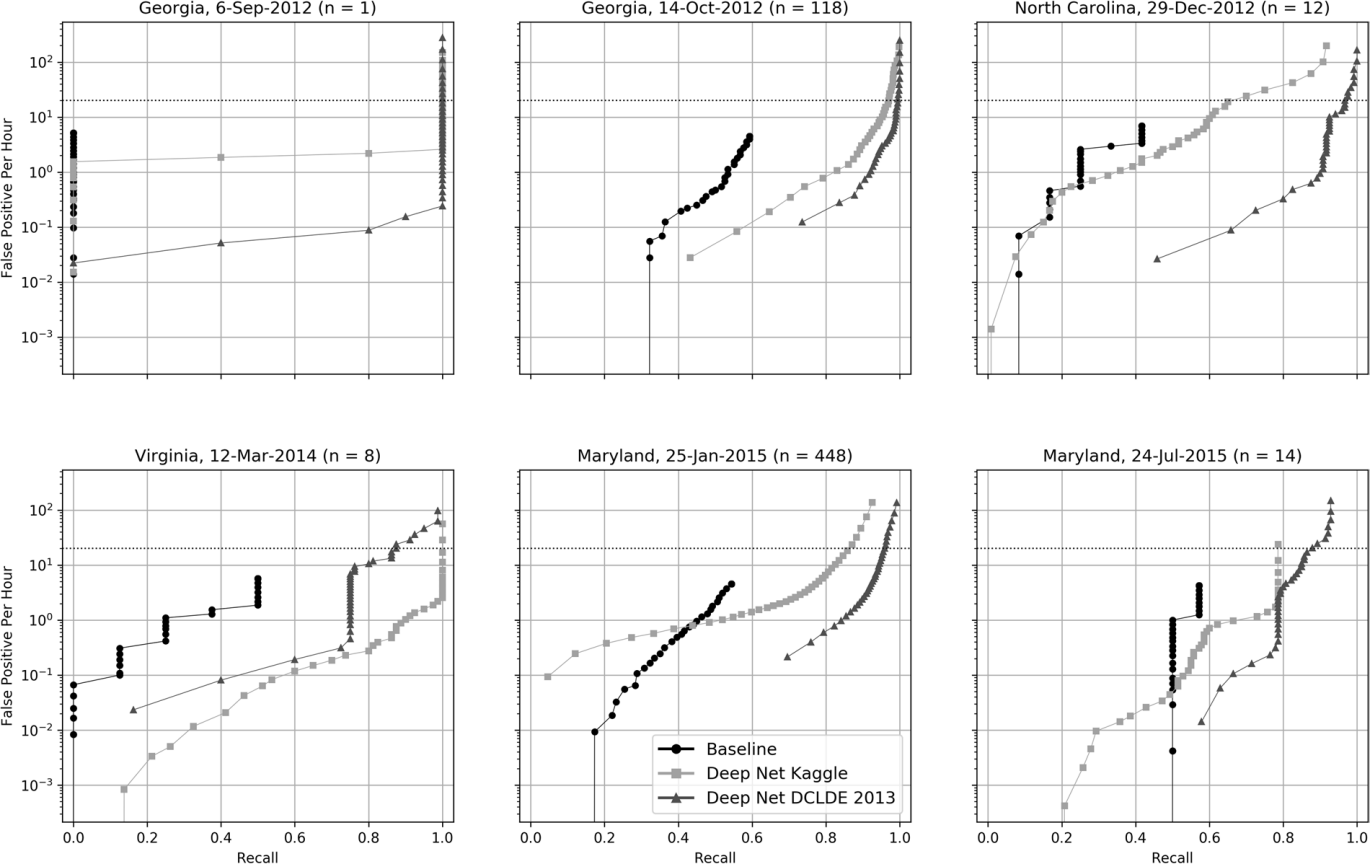
Recall vs. hourly false positive detections for generalization data from different geographic regions and dates. Results were averaged across 10 runs, each with random parameter initializations. The number of upcalls in the data on each of the six trial days is indicated by *n*.

To illustrate performance variability, we disaggregated false positives per hour by day and region (Fig. 2) and omitted the precision-recall curves, which had patterns similar to those generated by the first experiment. At nearly all recall rates, both deep nets yielded orders of magnitude fewer false positives than the baseline detector, suggesting that the deep net architecture, rather than differences in training data, was responsible for the performance gain. Both deep net models detected rare calls that the baseline method was unable to detect. An exception to this trend occurred on the day with the highest number of calls (25 January 2015). When thresholds were high (low recall), the baseline detector had fewer false positives per hour than the deep net trained with Kaggle data. As thresholds were lowered, the recall of the deep net became greater than that of the baseline, with similar or lower false positive rates.

In general, the deep net trained with DCLDE 2013 data outperformed the deep net trained with Kaggle data. The 12 March 2014 data from Virginia were an exception because the former detected the two of the eight upcalls with low scores, which have unconventional shape; one is an overlap between an upcall and another tonal sound, whereas the other has short duration and large bandwidth. In these data, at recall rates above 0.8, the number of false positives from the deep net trained with DCLDE 2013 data was roughly two orders of magnitude higher than those from the deep net trained with Kaggle data. However, on the whole, the performance curves obtained from the deep net trained with the DCLDE 2013 data were much higher than those produced by the deep net trained with Kaggle data. At a recall rate of 0.70, the deep net trained with the DCLDE 2013 data generated <0.30 false positives per hour across the entire data set. In contrast, the deep net trained with Kaggle data generated as many as 20 false positives per hour.

## Discussion

**Figure 3:**
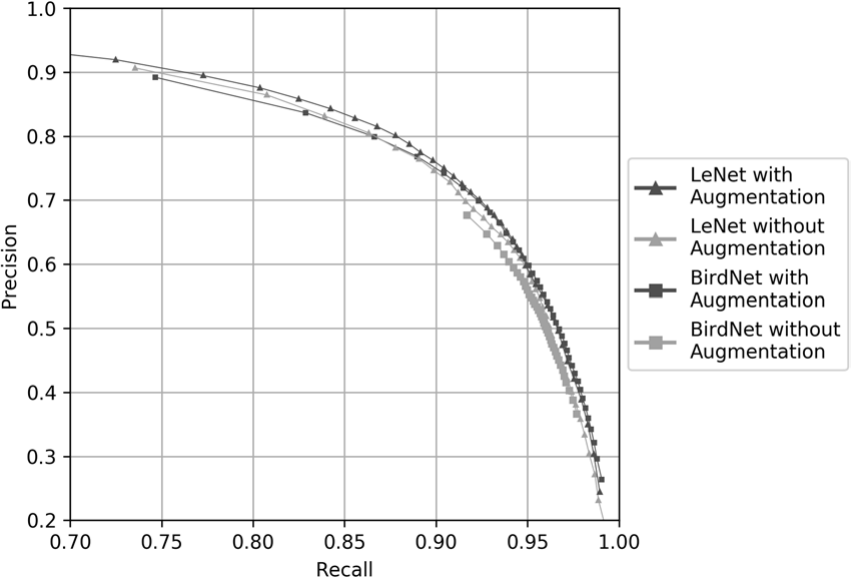
Precision-recall curves of LeNet and BirdNet with and without data augmentation. LeNet with and without data augmentation had average precisions of 0.903 and 0.898, respectively. BirdNet with and without data augmentation had average precisions of 0.891 and 0.830, respectively.

We used a variety of deep neural network architectures to detect the upcalls of endangered North Atlantic right whales. We enhanced learning through hard negative mining and data augmentation. Hard negative mining improved average precision (area under precision-recall curve) of LeNet from 0.852 to 0.903. Data augmentation enhanced the robustness of the detector to the variation of target signals and effect of noise (Fig. 3).

Deep neural networks had greater precision and recall than other contemporary methods and generated far fewer false positives. We found that deep networks can generalize well to recordings collected in different geographic regions and years that were not represented in the training data.

**Figure 4:**
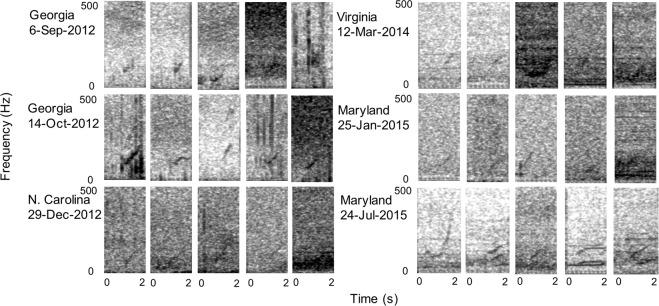
Examples of other signals which the deep net model predicted as right whale upcalls with probability >0.8. These calls are from data collected at times and places that were not represented in the training data. (See Figs. 5 and 7 for examples of right whale up calls).

Generalization tests showed that our detectors were able to recognize calls in different contexts than reflected in the training data and that the networks learned to recognize the characteristic upsweep of right whale upcalls. When we examined signals other than right whale upcalls in these data where the network had upcall predication probabilities of greater than 0.8, we saw that all such calls had upsweeping components (Fig. 4), suggesting that the network was learning to recognize the shape of the signal as opposed to the background noise. It also illustrates the difficulty of detecting right whale upcalls due to the presence of similar sounds.

An operating threshold must be selected when using an acoustic detector in an operational context (e.g., for conservation or mitigation). When monitoring rare or endangered species that vocalize infrequently, setting a low threshold reduces the number of missed calls and manual review subsequently eliminates false positives. In general, the greater the number of false positives per hour, the more expensive it becomes for analysts to verify the detections. This motivated us to report the number of false positives per hour as a function of recall.

In our experience, automated detection of right whale upcalls must generate fewer than an average of 20 false positives per recording hour (and channel) to be useful. An experienced analyst can verify up to 2,000 detections per working hour. At moderate to high recall values, validating false positive detections quickly dominates the analyst’s time. Consider one month of data from a single sensor and 600 known right whale upcalls. At an average rate of 20 false positives per hour, the data will contain approximately14,880 false positives and require 7.44 h of quality control by an analyst. Verifying up to 600 true positives will add less than 20 additional minutes of analyst time, a trivial incremental cost. Therefore, it is important to examine the number of false positives per hour when considering whether an automated detection process with analyst review is cost-effective in cases where the number of false positives far outnumbers the true number of calls.

At the rate of 20 false positives per hour, the best DCLDE 2013 detector retrieved 65% of the upcalls (Fig. 1) in the DCLDE validation data. Deep neural architectures retrieved from 85% to over 90% of the upcalls, or a >30% increase in the percentage of detections without increasing the false positives per hour. At the same false positives per hour across all days of the 2012–2015 generalization data (Fig. 2), the recall of the baseline detector with default parameters was 0.492. In contrast, our deep network produced recall values of 0.946 (DCLDE 2013 training) and 0.883 (Kaggle training). This represents recall improvements relative to the baseline detector of 92% and 79% respectively. For a given level of effort and financial cost, the deep neural network may be run at a lower relative threshold than the baseline model, increasing the likelihood of detecting and verifying the presence of rare species.

The deep nets that we considered may be applicable to ongoing passive acoustic monitoring efforts. For example, the Autobuoy call detection system^67^ in Massachusetts Bay is designed to reduce the likelihood of ship strike by alerting mariners of the presence of right whales and allowing them to take extra precautions when transiting the area (listenforwhales.org). An increase in recall may reduce the number of ship strikes.

Advances in automated sound detection allow rapid processing of large volumes of data, including animal vocalizations and anthropogenic sound that may be relevant to conservation decisions. Such information is especially valuable to research and management in marine ecosystems, in which visual detections of species can be considerably more challenging than in terrestrial ecosystems. Acoustic detection of such data can greatly improve understanding of habitat use, population biology, and animal behavior. Our work demonstrated that rapidly evolving deep learning techniques can directly advance the ability to detect a highly endangered marine mammal species.

## Materials and Methods

### Data collection

We leveraged data from three sources, the DCLDE 2013 workshop data, recordings collected throughout the range of the right whales from 2012–2015 by NOAA and BRP, and a Kaggle data competition (Table 1). The DCLDE 2013 workshop data were recorded in one week of 2009 and represent small temporal and spatial extents, but allow comparison to a number of contemporary algorithms that have been applied to these data. The recordings across the right whale range allow us to assess whether the algorithms generalize well to new data. When testing generalization, we trained separate models with the DCLDE 2013 and the Kaggle data to fairly compare the baseline algorithm that was trained with Kaggle data.

Both the DCLDE 2013 and the 2012–2015 data were collected with Marine Autonomous Recording Units (MARUs)^68^. MARUs were moored approximately 5 m above the sea floor with three 20.4 kg anchor plates. All MARUs were equipped with an HTI-94-SSQ hydrophone (High Tech, Inc., Long Beach, MS, USA) with a sensitivity of −169 dB re 1 V/µPa. MARUs had a flat frequency response (±3 dB) in the frequency range of right whale upcalls (15–585 Hz). After 23.5 dB pre-amplification, the effective analog system sensitivity was −145.5 dB re 1 V/µPa. The sensitivity of the ADC was 1 mV/bit. The dynamic range was 66.2 dB (85.5–151.7 dB re 1 µPa). The sample rate for all recordings was 2 kHz, and data were bandpass filtered between 10 and 800 Hz.

DCLDE 2013 data were recorded in a known right whale foraging area with 10 MARUs. DCLDE workshop data were derived from the first seven days of the deployment, 28 March through 3 April 2009, with the first four days designated as training data and the remaining days as validation data.

The 2012–2015 data were derived from MARU deployments within a portion of the right whale migratory corridor, including coastal Maryland, Virginia, North Carolina, and Georgia. Three to ten MARUs were deployed at each location, typically spaced 3–8 km apart.

**Figure 5:**
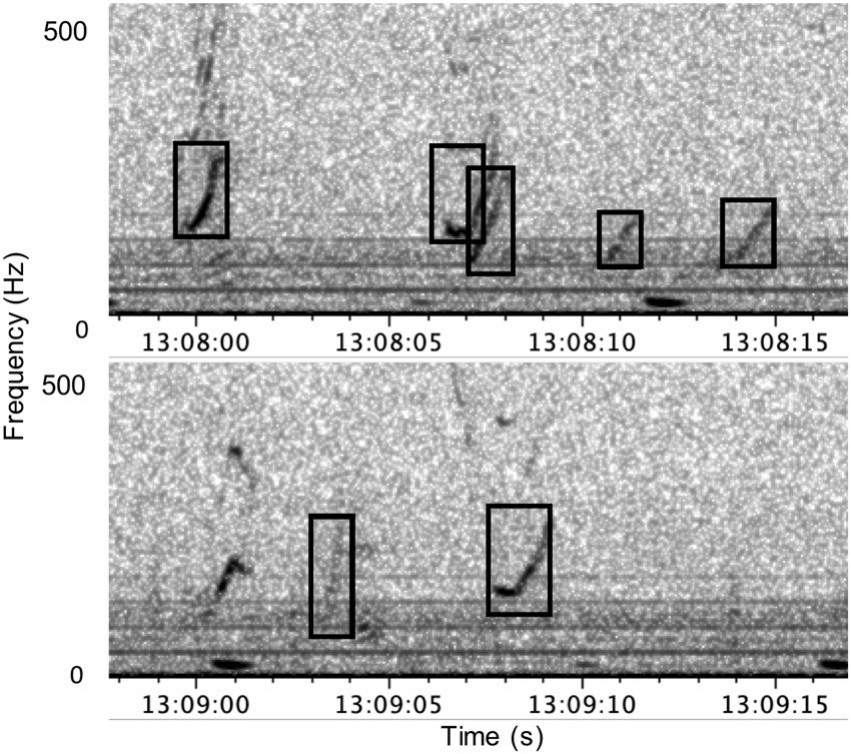
Analyst-identified upcalls in a spectrogram from DCLDE 2013 data collected on 29 March 2009 (2 kHz sample rate, discrete Fourier transform, 512 ms window, 51 ms advance, 3.9 Hz bins, Hann window). Upcall annotations (black boxes) indicate the approximate upper and lower frequency and the start and end times of the calls.

For all data sets, trained analysts visually inspected spectrograms to identify right whale upcalls. All upcalls observed by the analysists were annotated with time-frequency bounding boxes (Fig. 5). A second analyst confirmed the upcalls in the 2012–2015 data.

The Kaggle data were recorded with 10 auto-detection buoys (Autobuoy^67^) operated in the Boston Traffic Separation Scheme in Massachusetts Bay, which uses an onboard call detection system to continuously monitor the presence of right whale upcalls^53^. The detector was configured to run at a high recall (89% for calls with signal-to-noise ratio >9 dB), but also a high hourly false positives, accepting any tonal sounds that swept up in frequency, had a duration between 0.5 and 2 s and started their sweep between 55 and 157 Hz. Acoustic data were collected at a 96 kHz sample rate and 24 bit resolution. The hydrophone (HTI-96-MIN, High Tech, Inc., Long Beach, MS, USA) was suspended in the middle of the water column and had a sensitivity of −169.7 dB re 1 V/µPa. The Autobuoy had a flat frequency response (±3 dB) in the frequency range of right whale upcalls (15–585 Hz). After pre-amplification (6 dB), the effective analog system sensitivity was −163.7 dB re 1 V/µPa. The input clipping level of the analog-to-digital converter (ADC) was -/+1.4142 V.

Unlike DCLDE data and 2012–2015 MARU data, which are continuous sound streams, Kaggle data were selected among the output sound clips from the Autobuoy detector. When a potential upcall was detected, a 2 s recording was saved to onboard memory. This recording was down-sampled (2 kHz, 16 bit) and transmitted to a receiving station where analysts reviewed detections to assess their validity. A total of 30,000 2 s sound clips were used in the Kaggle data competition. 7,027 contained right whale upcalls, whereas the remainder represented a wide variety of false positives produced by the detector^53^, which include tonal sounds from other marine mammals such as humpback whales, anthropogenic sounds such as passing vessels, and self-noise from the Autobuoy. The goal of the Kaggle data competition in 2013 was to distinguish upcalls from other sounds.

### Data preparation

Training examples consisted of spectrograms (128 ms Hann window, 50 ms advance) of 2 s sound clips with a frequency range from 39.06 to 357.56 Hz, resulting in a 40 × 40 matrix of time-frequency magnitude values. We normalized the matrix by dividing each element by the sum of the squared elements in the matrix.

Analyst annotations of the DCLDE data were used to extract 6,916 positive (upcall present) examples of 2 s duration, starting 1 s prior to the temporal midpoint of each upcall. An initial set of 5,499 negative (upcall absent) examples was selected at random from periods during which upcalls were not present. A second set of 29,463 hard negative examples was acquired and added as part of the training data after applying a classifier trained with both the positive and the initial negative examples to the detection of training data and collecting the false positive detections. The same signal processing chain was used to create spectrograms for the Kaggle data except the random selection of negative examples. The negative examples of Kaggle data are part of the false-positive detections from the Autobuoy detection system.

To evaluate model performance, spectrograms of 2 s sounds were created every 0.1 s across the sound stream of the testing data.

### Deep neural network architectures

We explored two types of deep neural network architectures: convolutional neural networks (CNNs) and recurrent neural networks (RNNs)^69^. Convolutional networks begin with filters that are convolved with signals, producing new outputs that are weighted combinations of nearby elements of the signal. The size of the convolutional filter specifies a rectangular region of interest around each time-frequency bin of the spectrogram. Time-frequency nodes within the region of interest affect the output of the center time-frequency node that becomes an input to the next layer. Subsequent stages of CNNs combine the outputs of filters with non-linear functions, including those that select only the most active component (the max pooling operator). By contrast, RNNs use outputs from past or future inputs of the temporal sequence to inform the current prediction.

Both CNNs and RNNs generally employ a traditional feed-forward neural network as the last stage, which uses the previous outputs as features and produces a binary classification decision. Our networks output the probability how likely the input spectrogram contains an upcall. The learning process calculates a categorical cross-entropy loss function between the desired training output and the network’s current output. The gradient of this loss function is distributed backward through the network (back propagation), driving changes in each node’s weights and biases to reduce the loss. Repeated estimates of the loss function gradient and adjustment of model parameters implement a learning feedback loop.

We examined the ability of five deep neural network architectures to detect right whale upcalls in archival data and to generalize the learning to acoustic data from other locations, seasons, and years. The convolutional networks were LeNet^38^, BirdNET^34^, VGG, a very deep neural network^60^, ResNet, a deep residual network^61^, and Conv1D + GRU, a hybrid convolutional and recurrent neural network that uses one-dimensional convolutions and a gated recurrent unit^59^.

LeNet established many of the fundamental components of CNNs and we used the published architecture with minor modifications. We used max-pooling in place of average-pooling and tanh activation in place of rectified linear unit (ReLU) activation. We also applied dropout on the input layer as well as after the two max-pooling layers. BirdNET is one of few CNNs that identifies bird species in acoustic recordings through weakly supervised learning. We inserted a dropout layer with probability 0.2 immediately after each max-pooling layer and applied high L2 regularization (0.2) within convolutional layers in order to prevent overfitting. Conv1D + GRU was developed to tag environmental sounds. VGG learns complex features via filters with small kernel sizes and increased network depth. A challenge with back-propagation of the gradient is that the gradient diminishes with each layer^70^. ResNet mitigates this problem by allowing gradients to be propagated to previous layers through an identity function, facilitating the construction of very deep architectures.

**Figure 6:**
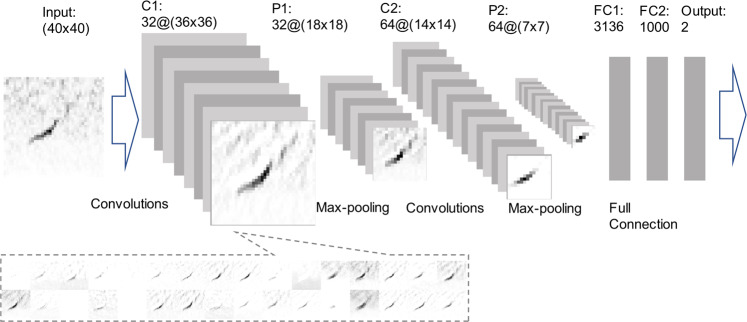
Representation of the LeNet convolutional neural network. C1 and C2 are feature maps generated by convolutions with output features of the C1 below. P1 and P2 are feature maps generated by subsampling through max pooling. FC1 and FC2 are vectors from fully connected layers. Feature maps of the C1 layer are shown below.

The power of deep convolutional architectures results from the large number of filters that learn to extract relevant features for detection (Fig. 6). Convolutions are executed on either input data (spectrograms or, in the case of Conv1D + GRU, a portion thereof) or the outputs of previous layers. Each output can be treated as a feature map. These outputs commonly are fused with subsampling techniques such as max pooling. For each filter, max pooling reduces the size of the feature map by a factor of four by selecting the maximum value for each non-overlapping 2 × 2 region. Max pooling reduces the resolution of the input feature map and provides an abstracted representation. The large number of filters of trainable weights and the large parameter space enable a good fit of the modeled input to the class labels. For example, LeNet applies 32 and 64 filters to the first and second sets of convolutions, respectively. Binary classification is applied to the fully connected layers generated from the second set of feature maps. Accordingly, the neural network acts as a detector where the classification decision is whether or not an upcall is present.

Overfitting is a serious challenge when developing deep neural networks given the high number of parameters in the network. In an extreme case, the deep neural network might remember all of the training data and yield 100% in-sample accuracy. However, given the model’s lack of generality, accuracy will be much lower when the model is applied to out-of-sample data. Many machine learning models incorporate one or more forms of regularization to prevent overfitting. For example, dropout minimizes overfitting by randomly omitting portions of the network during the training process^71^. We added three dropout layers to a LeNet model (Fig. 6), one each between P1 and C2, P2 and FC1, and FC2 and generation of the final output. Other architectures use batch normalization^72^, which renders zero-mean and unit standard variation of each layer’s inputs. BirdNET uses batch normalization and dropout in all layers except the input and output layers. Conv1D + GRU uses both batch normalization and dropout in the 1D convolutional layer and batch normalization on the two GRU layers. ResNet and VGG use batch normalization, but no dropout.

### Experiments

We conducted two experiments. First, we used the DCLDE 2013 data to assess and compare the performance of our deep neural architectures with the performance of detectors implemented by workshop participants. Second, we examined the ability of deep neural architectures to generalize to data collected years after the DCLDE 2013 data in different geographic regions.

We trained each architecture with custom software that used Python 3.5.2, Keras 2.2.4, and TensorFlow 1.5.0. We presented 1,000 data examples (i.e., a batch size of 1,000) to the model during each gradient estimate. We trained all models with 100 epochs, each representing an iteration through the full training data. These 100 epochs were sufficient for the accuracy metric of the training data to plateau. We used an adaptive moment optimizer (Adam^73^) with a learning rate decay of 0.005. To account for the difference between the number of positive and negative examples in the training data, we weighted the positive examples by a factor of three. We used ten instances of each model to analyze the effects of random initialization, and presented the mean results.

The DCLDE 2013 experiment followed the workshop protocol. Days one to four were used for classifier development, i.e., model training and model selection. Sound clips of both upcalls (positive class) and other sounds (negative class) were extracted from the four days of sounds. Days five to seven were used for testing, i.e., evaluating detection performance by consecutively applying the trained binary classifier every 0.1 s. We enhanced the training set with two methods, data augmentation^74^ and hard negative mining^75^. Data augmentation synthesizes additional examples to help with generalization. We randomly shifted positive and negative samples by up to + /− 200 ms, and pooled the shifted samples with the original examples to train a preliminary deep neural network. We used this deep net to predict potential detections (probability of call ≥0.5) across the entire four days of training data as described below.

**Figure 7:**
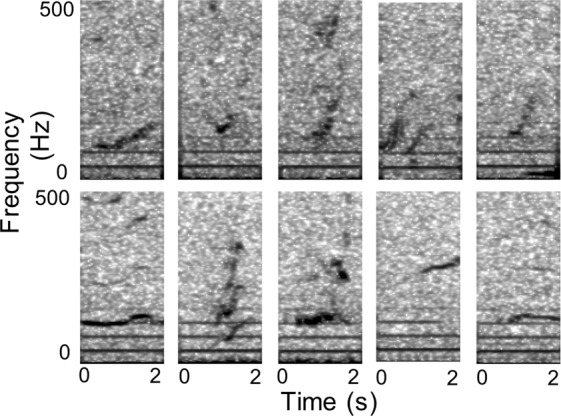
Spectrograms (2 kHz sample rate, discrete Fourier transform, 512 ms window, 51 ms advance, 3.9 Hz bins, Hann window) of examples used in training the neural networks. Top row: upcalls detected in the first round of training. Bottom row: false-positive detections identified as hard negative examples that we used to refine the detector.

We identified 14,371 false positives not already in the training set as difficult examples, henceforth *hard negatives*, and added them to the training pool from which a new deep net was trained. Including hard negatives (Fig. 7) is an essential step in the training process because doing so allows the network the opportunity to learn what signals are similar and subsequently discriminate them from true upcalls.

To determine how well the deep nets performed compared to existing technology, we obtained results of other algorithms from the DCLDE 2013 workshop and anonymized the results.

We used precision and recall to assess the performance of the trained model when applied to the withheld validation data. Precision is calculated as TP/(TP + FP), where TP is the number of true positives (correctly detected right whale upcalls) and FP is the number of false positives (erroneous detections). Recall is calculated as TP/(TP + FN), where FN is the number of false negatives (upcalls not detected). We also calculated the recall performance versus the number of false positives per hour, which is relevant to economic viability.

We evaluated trained deep nets on the three days of DCLDE 2013 testing data. We advanced a moving 2 s prediction window by 0.1 s at each step and computed the probability of right whale upcall presence for each model^69,76^. We used a non-maximum suppression^77^ process restricted to call probabilities ≥0.05 to remove overlapping predictions.

Our second experiment examined the ability of the deep nets to generalize to data collected under a variety of recording conditions. We compared the deep nets we developed here to a baseline right whale upcall detector that was part of the DCLDE 2013 challenge^64^, henceforth called the baseline detector. The baseline detector, which currently is deployed for monitoring right whales, incorporates 13 parameters that allow it to be tuned to specific environments. Here we used the default settings because we aimed to examine generalization behavior without adaptation. Data that we used to evaluate generalization were collected across multiple years and seasons, and throughout the range of the species.

We selected the best deep net from the first experiment because it was more likely to have overfitted to the environment from the week of recordings in Massachusetts Bay. The baseline detector was trained with Kaggle data, which created uncertainty in whether performance differences were due to training data or model architecture. Consequently, we trained two versions of the best deep net, one with the DCLDE 2013 data as described above and one with the Kaggle data. As in the first experiment, we trained 10 models with each data set.
