# Publisher Correction: Deep neural networks for automated detection of marine mammal species

**DOI:** 10.1038/s41598-020-67560-y

**Published:** 2020-06-30

**Authors:** Yu Shiu, K. J. Palmer, Marie A. Roch, Erica Fleishman, Xiaobai Liu, Eva-Marie Nosal, Tyler Helble, Danielle Cholewiak, Douglas Gillespie, Holger Klinck

**Affiliations:** 1 Center for Conservation Bioacoustics, Cornell Lab of Ornithology, Cornell University, Ithaca, NY 14850 USA; 2 Department of Computer Science, San Diego State University, San Diego, CA 92182 USA; 3 Department of Fish, Wildlife and Conservation Biology, Colorado State University, Fort Collins, CO 80523 USA; 4 Department of Ocean and Resources Engineering, University of Hawai’i at Mānoa, Honolulu, HI 96822 USA; 5 US Navy, Space and Naval Warfare Systems Command, System Center Pacific, San Diego, CA 92152 USA; 6 Northeast Fisheries Science Center, National Marine Fisheries Service, National Oceanic and Atmospheric Administration, Woods Hole, MA 02543 USA; 7 Sea Mammal Research Unit, Scottish Oceans Institute, University of St. Andrews, St Andrews, Fife, KY16 8LB Scotland

Correction to: *Scientific Reports* 10.1038/s41598-020-57549-y, published online 17 January 2020

In this Article, the legend for Table 1 is incorrect.

“Number of upcalls indicates the number of upcalls annotated by trained analysts. For deployments with two or more recorders, the number of upcalls indicates the total number of upcalls detected across all recorders. Shaded rows indicate data used to train neural networks. Non-shaded rows represent evaluation data. Negative examples for the Kaggle data represent the false detections flagged by the analysts as derived from non-right whale sources.”

should read:

“Data sources used to train and evaluate deep neural network performance. Number of upcalls indicates the number of upcalls annotated by trained analysts. For deployments with two or more recorders, it indicates the total number of upcalls detected across all recorders. Data from 28-Mar-09 to 31-Mar-09 were used to train neural networks. All others represent evaluation data. Negative examples for the Kaggle data represent the false detections flagged by the analysts as derived from non-right whale sources.”

In addition, the Acknowledgements section in this Article is incomplete.

“We are grateful to P. Dugan for running the BRP baseline detector; S. Kahl for sharing source code and advice on the methods; A. Rahaman, K. Hodge, B. Estabrook, D. Salisbury, M. Pitzrick, and C. Pelkie for helping with data analysis; F. Channell, C. Tessaglia-Hymes, and D. Jaskula for deploying and retrieving MARUs, and the DCLDE 2013 organizing committee. We thank the Bureau of Ocean Energy Management for the funding of MARU deployments, Excelerate Energy Inc. for the funding of Autobuoy deployment, and Michael J. Weise of the US Office of Naval Research for support (N000141712867).”

should read:

“We are grateful to P. Dugan for running the BRP baseline detector; S. Kahl for sharing source code and advice on the methods; A. Rahaman, K. Hodge, B. Estabrook, D. Salisbury, M. Pitzrick, and C. Pelkie for helping with data analysis; F. Channell, C. Tessaglia-Hymes, and D. Jaskula for deploying and retrieving MARUs, and the DCLDE 2013 organizing committee. We thank S. V. Parijs, G. Davis, C.W. Clark, L. Hatch, D. Wiley, and NOAA Fisheries for the DCLDE data and analyses. We thank the Bureau of Ocean Energy Management for the funding of MARU deployments, Excelerate Energy Inc. for the funding of Autobuoy deployment, and Michael J. Weise of the US Office of Naval Research for support (N000141712867).”

