# Author Correction: Deep neural networks for automated detection of marine mammal species

**DOI:** 10.1038/s41598-021-00460-x

**Published:** 2021-10-21

**Authors:** Yu Shiu, K. J. Palmer, Marie A. Roch, Erica Fleishman, Xiaobai Liu, Eva-Marie Nosal, Tyler Helble, Danielle Cholewiak, Douglas Gillespie, Holger Klinck

**Affiliations:** 1 Center for Conservation Bioacoustics, Cornell Lab of Ornithology, Cornell University, Ithaca, NY 14850 USA; 2 Department of Computer Science, San Diego State University, San Diego, CA 92182 USA; 3 Department of Fish, Wildlife and Conservation Biology, Colorado State University, Fort Collins, CO 80523 USA; 4 Department of Ocean and Resources Engineering, University of Hawai’i at Mānoa, Honolulu, HI 96822 USA; 5 System Center Pacific, US Navy, Space and Naval Warfare Systems Command, San Diego, CA 92152 USA; 6 Northeast Fisheries Science Center, National Marine Fisheries Service, National Oceanic and Atmospheric Administration, Woods Hole, MA 02543 USA; 7 Sea Mammal Research Unit, Scottish Oceans Institute, University of St. Andrews, St. Andrews, Fife, KY16 8LB Scotland

Correction to: *Scientific Reports* 10.1038/s41598-020-57549-y, published online 17 January 2020

The original version of this Article contained errors.

Table 1 omitted to reference the experimental data and its funding sources. As the result, References 78-83 were omitted from Table 1. Added References are listed below:

Hatch, Leila T., et al. Quantifying loss of acoustic communication space for right whales in and around a US National Marine Sanctuary. *Conservation Biology* 26.6, 983-994 (2012).

Clark, C.W., et al. An ocean observing system for large-scale monitoring and mapping of noise throughout the Stellwagen Bank National Marine Sanctuary. Cornell University, Ithaca, NY (2010).

Cholewiak, D., et al. Communicating amidst the noise: modeling the aggregate influence of ambient and vessel noise on baleen whale communication space in a national marine sanctuary. *Endangered Species Research*, *36*, 59-75. (2018).

Rice, A. N. *et al.* Baseline bioacoustic characterization for offshore alternative energy development in North Carolina and Georgia wind planning areas. U.S. Department of the Interior, Bureau of Ocean Energy Management, Gulf of Mexico OCS Region., New Orleans, LA. (2015).

Salisbury, D. P., Estabrook, B. J., Klinck, H., & Rice., A. N. Understanding marine mammal presence in the Virginia offshore wind energy area. US Department of the Interior, Bureau of Ocean Energy Management, Sterling, VA. (2019)

Bailey, H. *et al.* Determining offshore use by marine mammals and ambient noise levels using passive acoustic monitoring. U.S. Department of the Interior, Bureau of Ocean Energy Management., Sterling, VA. (2018)

Consequently, the legend of Table 1 has been corrected accordingly,

“Number of upcalls indicates the number of upcalls annotated by trained analysts. For deployments with two or more recorders, the number of upcalls indicates the total number of upcalls detected across all recorders. Shaded rows indicate data used to train neural networks. Non-shaded rows represent evaluation data. Negative examples for the Kaggle data represent the false detections flagged by the analysts as derived from non-right whale sources.”

now reads:

“Data sources used to train and evaluate deep neural network performance. Number of upcalls indicates the number of upcalls annotated by trained analysts. For deployments with two or more recorders, the number of upcalls indicates the total number of upcalls detected across all recorders. Shaded rows indicate data used to train neural networks. Non-shaded rows represent evaluation data. Negative examples for the Kaggle data represent the false detections flagged by the analysts as derived from non-right whale sources. Contract grants: (i) Office of Naval Research grant (number N00014–07-1–1029) awarded by the National Oceanographic Partnership Program; (ii) U.S. Department of the Interior, Bureau of Ocean Energy Management grant (number M10PC00087); (iii) U.S. Department of the Interior, Bureau of Ocean Energy Management grant (number M15AC00010); (iv) U.S. Department of the Interior, Bureau of Ocean Energy Management grant (number M14AC00018); Maryland Department of Natural Resources grants (14-14-1916, 14-17-2241)”

The original Table 1 and accompanying legend appear below.

**Table 1:**
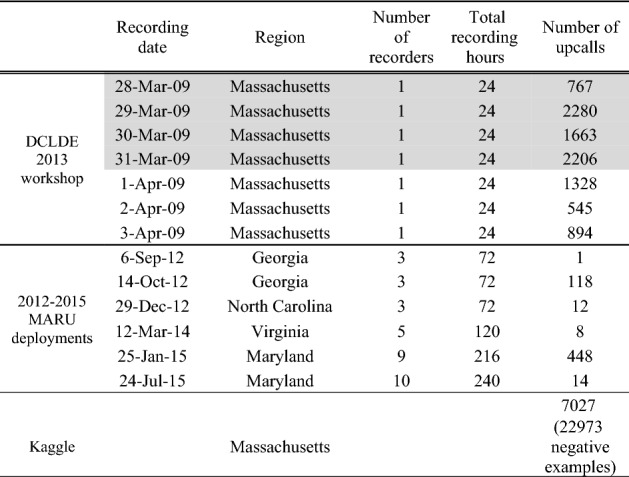
Number of upcalls indicates the number of upcalls annotated by trained analysts. For deployments with two or more recorders, the number of upcalls indicates the total number of upcalls detected across all recorders. Shaded rows indicate data used to train neural networks. Non-shaded rows represent evaluation data. Negative examples for the Kaggle data represent the false detections flagged by the analysts as derived from non-right whale sources.

Finally, in the Acknowledgments,

“We are grateful to P. Dugan for running the BRP baseline detector; S. Kahl for sharing source code and advice on the methods; A. Rahaman, K. Hodge, B. Estabrook, D. Salisbury, M. Pitzrick, and C. Pelkie for helping with data analysis; F. Channell, C. Tessaglia-Hymes, and D. Jaskula for deploying and retrieving MARUs, and the DCLDE 2013 organizing committee. We thank the Bureau of Ocean Energy Management for the funding of MARU deployments, Excelerate Energy Inc. for the funding of Autobuoy deployment, and Michael J. Weise of the US Office of Naval Research for support (N000141712867).”

now reads:

“We are grateful to P. Dugan for running the BRP baseline detector; S. Kahl for sharing source code and advice on the methods; A. Rahaman, K. Hodge, B. Estabrook, D. Salisbury, M. Pitzrick, and C. Pelkie for helping with data analysis; F. Channell, C. Tessaglia-Hymes, and D. Jaskula for deploying and retrieving MARUs, and the DCLDE 2013 organizing committee. We thank S. V. Parijs, G. Davis, C.W. Clark, L. Hatch, D. Wiley, and NOAA Fisheries for the DCLDE data and analyses. We thank the Maryland Department of Natural Resources secured the funding for the data collection offshore of Maryland from the Maryland Energy Administration’s Offshore Wind Development Fund (14-14-1916, 14-17-2241). We thank the Bureau of Ocean Energy Management for funding MARU deployments and data collection (M10PC00087 for Georgia and North Carolina, M15AC00010 for Virginia, M14AC00018 for Maryland), Excelerate Energy Inc. for the funding of Autobuoy deployment, and Michael J. Weise of the US Office of Naval Research for support (N000141712867).”

The original Article has been corrected.

